# PPR-SSM: personalized PageRank and semantic similarity measures for entity linking

**DOI:** 10.1186/s12859-019-3157-y

**Published:** 2019-10-29

**Authors:** Andre Lamurias, Pedro Ruas, Francisco M. Couto

**Affiliations:** LASIGE, Departamento de Informática, Faculdade de Ciências, Universidade de Lisboa, Lisboa, 749-016 Portugal

**Keywords:** Ontologies, Text mining, Entity linking, Biomedical literature, ChEBI, HPO, GO

## Abstract

**Background:**

Biomedical literature concerns a wide range of concepts, requiring controlled vocabularies to maintain a consistent terminology across different research groups. However, as new concepts are introduced, biomedical literature is prone to ambiguity, specifically in fields that are advancing more rapidly, for example, drug design and development. Entity linking is a text mining task that aims at linking entities mentioned in the literature to concepts in a knowledge base. For example, entity linking can help finding all documents that mention the same concept and improve relation extraction methods. Existing approaches focus on the local similarity of each entity and the global coherence of all entities in a document, but do not take into account the semantics of the domain.

**Results:**

We propose a method, PPR-SSM, to link entities found in documents to concepts from domain-specific ontologies. Our method is based on Personalized PageRank (PPR), using the relations of the ontology to generate a graph of candidate concepts for the mentioned entities. We demonstrate how the knowledge encoded in a domain-specific ontology can be used to calculate the coherence of a set of candidate concepts, improving the accuracy of entity linking. Furthermore, we explore weighting the edges between candidate concepts using semantic similarity measures (SSM). We show how PPR-SSM can be used to effectively link named entities to biomedical ontologies, namely chemical compounds, phenotypes, and gene-product localization and processes.

**Conclusions:**

We demonstrated that PPR-SSM outperforms state-of-the-art entity linking methods in four distinct gold standards, by taking advantage of the semantic information contained in ontologies. Moreover, PPR-SSM is a graph-based method that does not require training data. Our method improved the entity linking accuracy of chemical compounds by 0.1385 when compared to a method that does not use SSMs.

## Background

Entity linking matches each entity mention in a document to an entry of a knowledge base (KB) that unequivocally represents that concept [1, 2]. This task is a fundamental component of text mining systems, in order to integrate the information described in the literature across multiple documents [3]. Entity linking has been applied to accomplish various objectives, for example, to link persons to their family members [4], to enrich a domain-specific KBs with information from Wikipedia tables [5], or to link events described in tweets [6]. Entity linking is fundamental to real-world text mining applications where the same concept may be referred to in different spellings and lexical variations across various documents. Due to the diverse nomenclature of biomedical entities, it is often a challenge to match these to KB entries. While several biomedical Named Entity Recognition (NER) approaches have been developed to recognize, for example, genes, drugs and diseases entities in documents [7, 8], fewer approaches exist to link these entities to a KB, given its higher complexity.

Entity linking can be incorporated into a NER system to perform both tasks at once. For example, by directly matching a list of concept names and synonyms from a controlled vocabulary to the text, it is possible to directly obtain the respective identifiers. However, this approach will be restricted to the names and synonyms considered in the KB, even when string matching algorithms can be used to deal with misspellings and other lexical variations. As most state-of-the-art NER systems are based on machine learning algorithms, they focus on recognizing segments of text that refer to entities of interest, requiring an additional method to match each named entity to a KB. Entity linking can also be modeled as a ranking task, where a list of candidate matches of an entity is ordered from highest to lowest confidence level. The input data consists of a graph, where nodes represent associations between named entities and candidate matches obtained from the KB. The edges represent links between concepts found in an external database. The objective of this approach is to select the set of candidate matches that maximizes the global coherence between entities.

In biomedicine, ontologies are commonly used to organize knowledge about a specific domain, providing a formal representation of concepts and their relations according to the domain. As such, they can be used as reference KBs for text mining tasks such as entity linking [9, 10]. For example, an ontology enables us to calculate the semantic similarity between two concepts and compare which concepts have more in common. Therefore, this source of information can be incorporated into entity linking approaches to improve their performance.

**Fig. 1:**
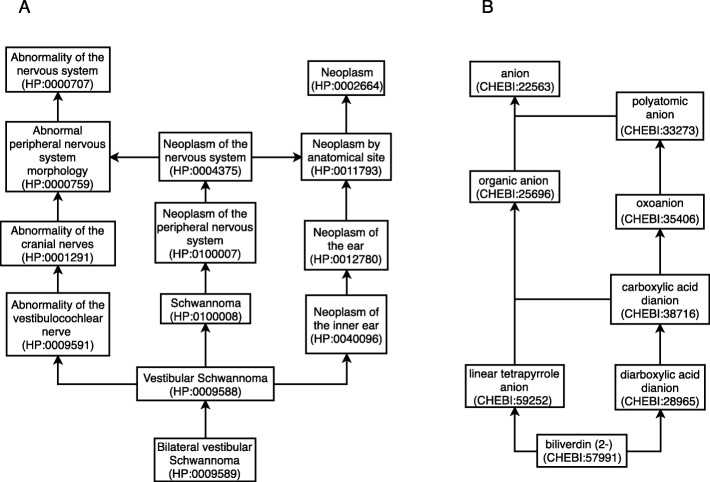
Excerpts of the ontologies used in this work. Arrows indicate *is-a* relations. Each concept may have more than one ancestor as well as multiple descendants. **a** HPO; **b** ChEBI

The Human Phenotype Ontology (HPO) [11] provides a standardized vocabulary to describe phenotypic variations in human disease which can be used by computational applications. This ontology was first released in 2008, originally composed by 8000 concepts, with the latest version containing 11,000 concepts. Figure 1a shows an excerpt of this ontology, with focus on the ancestors of the concept “Bilateral vestibular Schwannoma” (HP:0009589). The arrows describe *is-a* relations, providing a detailed classification of each concept. Chemical Entities of Biological Interest (ChEBI) contains over 46,000 molecular entities, with each entity being organized in an ontology structure [12]. The ChEBI ontology contains *is-a* relations, but also other types of relations such as *has part*, *has role*, and chemistry specific relations such as *is conjugate base of* and *is tautomer of*. Figure 1b shows the closest *is-a* ascendants of the ChEBI concept “biliverdin (2-)”.

Entity linking is a challenging task for biomedical literature when compared to other domains. For example, while there is no exact match for "iron chloride" in ChEBI, a database of chemical entities with biological interest [13], there are 157 abstracts on PubMed that match that exact string at the time we were writing this manuscript. These cases are problematic to automatic approaches because the entity string itself is ambiguous, requiring more advanced approaches to resolve this ambiguity. According to the Human Phenotype Ontology (HPO), dyschromatopsia and color-blindness refer to the same phenotype. Therefore a search for one of those names should retrieve documents that also mention the other one. Another example, a protein may be mentioned by its full name or by an acronym; in this case, the normalization process should assign the same identifier to both occurrences. To properly perform biomedical entity linking, it is necessary to account for these issues, as well as with the constant flow of newly published information.

PageRank is a graph-based algorithm initially developed to rank web pages for search results [14]. An adaptation of this algorithm, Personalized PageRank (PPR) [15], has been successfully applied to Word Sense Disambiguation [16] and Named Entity Disambiguation [17], which are tasks similar to entity linking [18]. The PPR algorithm, which we make use of in this work, is based on random walks along the graph, with a given probability of jumping to a specific source node.

Our main contribution is PPR-SSM, a novel domain-specific entity linking method for documents annotated with named entities that can be applied to various domains. Our method uses the PPR algorithm on a graph obtained from the relations established in the ontology, and explores the semantic similarity between the candidate matches of each entity to maximize the global coherence. We applied this method to three gold standards: i) one annotated with chemical entities; ii) one annotated with human phenotypes; and iii) annotated with gene ontology concepts. We used the ChEBI, HPO, and GO ontologies as our domain-specific KBs in the chemical, phenotype, and gene ontology gold standards, respectively. This method outperformed string matching and other PPR approaches. We also studied the effect of different semantic similarity measures in the results. We provide the code used in the experiments^1^, along with usage examples and a Docker image.

### Related work

Previous studies follow mainly two types of approaches: local similarity approaches, where the similarity between the entity text and candidate match is explored, and global approaches, which attempt at selecting the set of candidate matches that best represents the entities of a document [19, 20]. One of the most commonly used KBs for entity linking is Wikipedia, which contains information about a great variety of topics. For this reason, it can be used to map entities of different domains to a KB. This variety of topics also increases the difficulty of the task, since the same expression can have different meanings according to its context. The disambiguation pages show the diverse meanings that an expression may have. For example, “New York” can refer to the state, the city in the state of New York, cities in other states, works of art, sports teams and ship names.

Bunesco et al. presented a method based on Support Vector Machines, using a dictionary generated from Wikipedia to detect and link entities [21]. Other authors aimed at maximizing the global coherence between the linked entities [22–24]. Pershina et al. presented a graph-based method based on the Personalized PageRank (PPR) algorithm to this task, incorporating both local and global coherence [25]. They assumed that the probability of each node is related to how likely it is to fit with the other highest scoring nodes. More recently, Radhakrishnan et al. presented a method that improved entity similarity by training embedding vectors on a densified KB [20]. Since the majority of entity linking gold standards are based on Wikipedia, these systems are developed for general KBs, and rarely focus on domain-specific KBs.

#### Graph-based approaches

Several graph-based approaches have been proposed for entity linking. [26] developed a graph-based framework to rank the entries of a database according to their relevance to a query. [27] proposed a method to rank the concepts and relations of an ontology according to their importance to the domain. Although this method is helpful to better understand a domain using ontologies, the authors did not explore its utility for other text mining tasks. [28] explored Markov networks for entity linking, applied to citation databases. These types of approaches require training data, which is not always available, particularly in some biomedical domains. Unlike other authors that explored graph-based methods for entity linking, we propose a method that takes advantage of the semantic relations described in the KB.

#### Biomedical entity linking

Wikipedia as a KB for entity linking has two properties that are useful for this task: redirect pages, which account for synonyms and lexical variations; and disambiguation pages, which account for strings with multiple meanings. While biomedical ontologies can incorporate synonyms, there is no equivalent to disambiguation pages. When such ambiguity arises, it is necessary to understand the context of the sentence to determine the correct definition.

The gene normalization task of BioCreative consisted in determining the unique identifiers of genes and proteins mentioned in scientific articles [29, 30]. The objective of this task, as with the other BioCreative tasks, was to promote the development of new text mining methods specifically for biomedical text. The organizers selected and manually annotated articles with gene names, using Entrez Gene as reference. Three editions of this task were organized, each edition increasing the difficulty, with the final edition requiring the full-text annotation and being species non-specific. The gold standards developed for this task were made available and can then be used to benchmark new methods. Tsuruoka et al. [31] presented a method to develop heuristic rules for biomedical entity linking automatically. Their method obtained better computational performance than string matching while requiring minimal expert knowledge in the development of the rules.

A domain-specific ontology can be defined as a directed acyclic graph where each node represents a concept of the domain and the edges represent known relations between these concepts [32]. This definition is the traditional representation of existing biomedical ontologies, which are nowadays a mainstream approach to formalize knowledge about entities, such as genes, chemicals, phenotypes, and disorders. Biomedical ontologies are usually publicly available and cover a large variety of topics of Life and Health Sciences. The success of exploring a given biomedical ontology for performing a specific task can be easily extended to other topics due to the standard structure of biomedical ontologies. For example, the same measures of metadata quality have been successfully applied to resources annotated with different biomedical ontologies [33]. Zheng et al. [34] developed a graph-based approach to biomedical entity linking, by performing collective inference over a text window and using entropy to estimate the importance of each edge. While their method is similar to ours in some ways, we explore the ontology even further by incorporating the information content of each concept and its similarity to the other candidate concepts. Our method combines the advantages of PPR-based methods that do not require training data, with domain knowledge from biomedical ontologies. Therefore, it can be adapted for other domains, as long as there is an exhaustive and domain-specific ontology available.

## Results

### Data

We evaluated our method on three gold standards, consisting of biomedical documents manually annotated with ontology concepts. Table 1 presents a comparison between the gold standards. The ChEBI-patents corpus consists of 40 patent documents annotated with chemical entities, using the ChEBI ontology as reference. This gold standard was developed by a team of curators from ChEBI and the European Patent Office. The documents were selected to be representative of the universe of chemical patent documents. Whenever possible, the curators added the ChEBI concept identifier to the entity annotations. Since we were interested only in linking entity mentions to concept identifiers, we discarded entity mentions that were not assigned an identifier. There were 8407 textual entity mentions annotated with ChEBI identifiers in this corpus, corresponding to 2081 unique entity mentions. The ChEBI team provides an API that can be used to retrieve a list of concepts associated with a text search, which we used to obtain the candidate list for each entity. Since the annotation process was performed in 2009, we also used the ChEBI API to update concept identifiers that have changed since then automatically.

**Table 1:** Summary of the gold standards used for evaluation

Gold standard	ChEBI-patents	HPO-GSC	CRAFT-BP	CRAFT-CC
Documents	40	228	67	67
Total entities	18061	2773	9280	4075
w/ ID	8407	2773	9280	4075
w/ candidates	6607	1890	9280	4075
Entities/doc	210.2	12.2	138.5	60.8

Additionally, we evaluated our method on a gold standard corpus of 228 scientific abstracts annotated with human phenotypes, associated with the Human Phenotype Ontology (HPO), which we refer to as HPO-GSC. We used an updated version of this corpus, which aimed at improving the consistency of the annotations [35]. A total of 2773 textual named entities were annotated in this corpus, corresponding to 2170 unique entity mentions. We found that phenotype entities were more varied regarding nomenclature due to the existence of more synonyms for the same phenotype when compared to chemical entities. Comparing with the ChEBI-patents corpus, we can see that this corpus has fewer entities per document (ChEBI-patents: 210 entities/document; HPO-GSC: 12 entities/document). This ratio is relevant for our method because it aims at maximizing the coherence between entities, and documents with fewer entities are more prone to errors. We obtain a list of candidates for each entity through fuzzy string matching with the labels and synonyms of the HPO.

In order to compare our work with the state-of-the-art, we used the Colorado Richly Annotated Full-Text (CRAFT) Corpus with articles annotated with Gene Ontology (GO) concepts [36]. The v3.1 release includes 67 articles from PubMed Central Open subset. The articles in this corpus were independently annotated with concepts belonging to the three GO sub-ontologies: Biological Process (BP), Cellular Component (CC) and Molecular Function (MF). The annotations pertaining to MF subset were not used due to the low number of concepts annotated and the high number of concept repetition. The subsections of the corpus containing files annotated with BP and CC concepts are thus referred as CRAFT-BP and CRAFT-CC, respectively. There were 4075 named entities annotated in CRAFT-CC and 9280 in CRAFT-BP.

**Fig. 2:**
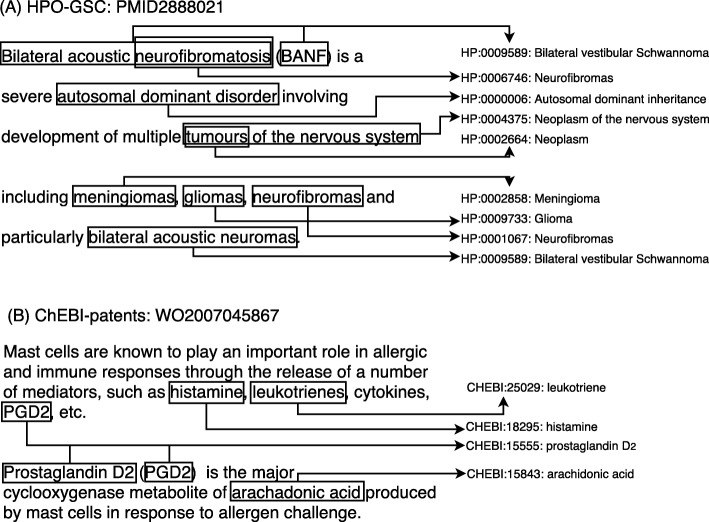
Example of the annotations associated with each of the gold standard used. For each entity mention, surrounded by a rectangle, we show its ontology ID and label. **a** HPO-GSC; **b** ChEBI-patents

Figure 2 shows an excerpt of the ChEBI-patents and HPO-GSC corpora, to demonstrate the type of information that was annotated by the curators. While in some cases the label of the concept matches the textual mention, in other cases there are some differences. Acronyms are common to both phenotypes and chemical entities. The HPO-GSC gold standard contains some overlapping entities, which could be mapped to different ontology concepts. While “neurofibromatosis” and “neurofibromas” were mapped to different concept identifiers, the current version of HPO merged those two concepts. As with the ChEBI-patents gold standard, we retrieved the most recent identifier of each concept annotated on each gold standard. Other challenges consist in dealing with plurals (both the entity text and concept label can be plural or singular) and abbreviations and acronyms (the ontology may have some of these synonyms but not all).

We used the April 2018 release of ChEBI, the March 2018 release of HPO and the February 2019 release of GO. The version of the ChEBI ontology that was used has about 54k manually verified chemical compounds. This ontology is curated by experts and updated monthly, while various sources are used to keep it as complete as possible, including user submissions. The HPO contains about 13k phenotypes and is focused on medically relevant phenotypes, and associating those phenotypes with diseases. This ontology is used in various applications that deal with clinical data. The GO is a structured representation for gene and gene product functions across many different organisms, like humans or bacteria, and is divided in three sub-ontologies as previously referred: Biological Process (BP) includes concepts describing biological pathways, for example, “cell death” (GO:0008219); Molecular Function (MF) includes concepts related with elemental functions such as “catalytic activity” (GO:0003824), being normally the “building blocks” of the biological pathways described by BP concepts; Cellular Component (CC) concepts refer to the place where above events occur, like “cytoplasm” (GO:0005737). The referred GO version includes 45,023 concepts, of which 29,699 belong to BP sub-ontology and 4,211 belong to CC sub-ontology. These ontologies tackle specific and complex areas of knowledge that benefit greatly from text mining methods.

### Evaluation setup

**Fig. 3:**
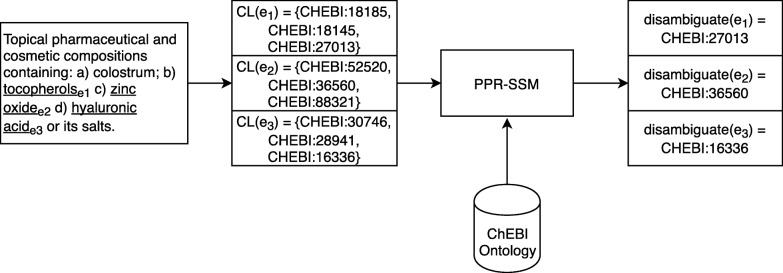
Pipeline of the PPR-SSM method to the ChEBI-patents corpus. For each entity, a candidate list *CL*(*e*) is obtained. The proposed ontology-based scoring function is applied to each element of the list, and the top scoring element is chosen

We evaluated each model considering the entities that were manually mapped to an ontology concept and for which the correct solution was in the set of candidates. Using the matching methods presented in the Methods section, we obtain a list of candidates for each entity. Table 1 shows that on the datasets used, most entities had its solution in the respective candidate list. Figure 3 shows an example of how we applied our method to the ChEBI-patents corpus.

We found that many concepts were not directly linked to each other in the ontology, meaning that the graph of each document would not have enough edges to apply PPR. For this reason, we studied the effect of considering the transitivity of subsumption relations, with a maximum distance threshold between 1 and 8. For example, if 5 is the maximum distance allowed, we would consider that there is an edge in the graph between two nodes if the shortest path between the respective concepts in ontology is equal to or less than 5 edges.

We use the scoring functions described in (3) and (12) to rank the candidate list of each entity mention. In case of a tie, we pick the candidate with more subclasses. We considered only candidates with a matching score higher than 0.7 for CHEBI-patents and HPO-GSC entities and higher than 0.6 for CRAFT-BP and CRAFT-CC entities which were determined empirically to be the best threshold values. We then compared the predicted candidate with the gold standard to calculate the accuracy score.

The PPR algorithm was computed using the Monte Carlo approach presented by Fogaras and Racz [15]. We executed 2,000 iterations for each source node, performing five steps of PPR, with a probability of jumping to source node equal to 0.2. These values were suggested by Pershina et al. [25], which we kept since we saw no major improvements with a different number of iterations, steps or jump probability.

### Experiments

Table 2 compares the accuracy of the proposed method with a string matching baseline and two other versions of the PPR algorithm: the first consisting of the PPR-based approach proposed by Pershina et al. adapted to biomedical domain-specific ontologies and the second adding a weight to the contribution of each node based on its Information Content (IC) (1). We performed a baseline evaluation, which consisted in picking the top candidate with highest string matching similarity. Adding semantic similarity as a factor in the contribution of each candidate has a positive effect, obtaining a higher accuracy than the other approaches.

**Table 2:** Accuracy of PPR-SSM compared with a baseline and PPR model, on the ChEBI-patents, HPO-GSC, and CRAFT (Biological Process and Cellular Component) gold standards

Method	ChEBI-patents	HPO-GSC	CRAFT-BP	CRAFT-CC
Top match	0.5271	0.6380	0.7744	0.6899
PPR	0.6654	0.5544	0.6923	0.6166
PPR-IC	0.8026	0.6557	0.8204	0.7247
PPR-SSM	0.8039	0.6825	0.8244	0.7258

**Fig. 4:**
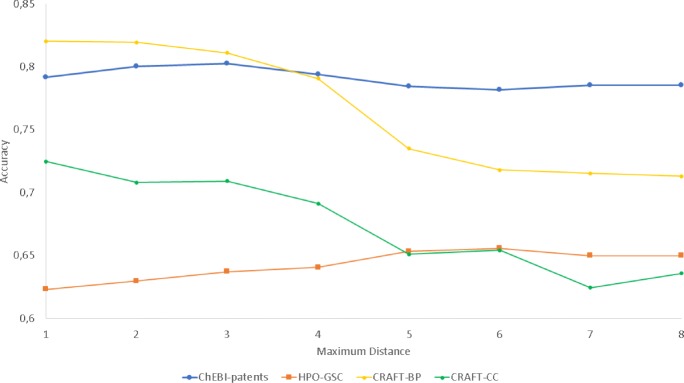
Comparison of different maximum distance values using the PPR-IC approach. For the CRAFT corpora (both BP and CC), the maximum accuracy is achieved using only direct links between concepts, while ChEBI and HPO-GSC benefit from including longer links

Figure 4 shows the effect of different maximum distance thresholds between concepts of the ontology. We compared paths of length 1, which means that there is a direct relationship between two concepts, to length 8, meaning that if there is a path shorter or equal to 8 between the concepts in the ontology, an edge is created in the graph. Each gold standard has a different optimal distance, with ChEBI-patents obtaining its best accuracy with distance 3, HPO-GSC with distance 6 and CRAFT-BP and CRAFT-CC both with distance 1. According to our experiments, the concepts linked by distances greater than those values do not contribute positively to the estimation of coherence within candidates. We used those distance values when comparing different PPR-based approaches (Table 2) and Semantic Similarity Measures (SSM) (Table 3).

**Table 3:** Comparison of different semantic similarity measures for PPR-based entity linking

SSM	IC_shared_	ChEBI-patents	HPO-GSC	CRAFT-BP	CRAFT-CC
Resnik	MICA	0.7916	0.6306	0.7444	0.6439
	DCA	0.7916	0.6340	0.7545	0.6439
Lin	MICA	0.7965	0.6825	0,8244	0.7190
	DCA	0.7965	0.6775	0.8216	0.7258
JC	MICA	0.8014	0.6775	0.8177	0.6985
	DCA	0.8039	0.6633	0.8199	0.6997

We used three SSMs for our PPR-SSM model: Resnik, Lin, and Jiang-Conrath (JC). Furthermore, we compare for each SSM the usage of Most Informative Common Ancestors (MICA) and Disjunctive Common Ancestors (DCA) to calculate the shared information content. Table 3 shows the results of this comparison.

Comparing the results obtained with each SSM, we can see that different measures obtain the best results on each gold standard. While JC-DCA obtains the best accuracy on the ChEBI-patents, Lin-MICA obtains the best accuracy on HPO-GSC and on CRAFT-BP and Lin-DCA on CRAFT-CC. In all cases, the Resnik measure obtains lower scores than the PPR-IC model. The main difference between Resnik and the other measures is that it does not take into account the individual IC of the two concepts. On ChEBI-patents, none of the measures had a noticeable effect on the performance, and in most cases, it decreases the accuracy. However, the PPR-IC model leads to considerable improvement, so there would be fewer and more difficult cases for the PPR-SSM model to resolve. A similar situation occurred for both CRAFT-BP and CRAFT-CC, despite the more modest increase in disambiguation accuracy by PPR-IC model comparing to ChEBI-patents. As the effect of the PPR-IC model on HPO-GSC was not as high, both Lin and JC measures improved the results. These findings seem to suggest that SSM can increase the disambiguation accuracy especially in cases where the PPR-IC model does not improve accuracy substantially. If the accuracy is already high, the SSM will have less impact on its improvement.

## Discussion

We manually analyzed the errors of the PPR-SSM model on each gold standard, in order to understand the limitations of our approach. On the ChEBI-patents corpus, some errors were due to the same words being used to refer to a family of compounds and a type of chemical compound. For example, “polyamine” can refer to CHEBI:51349 (polyamine macromolecule) and CHEBI:88061 (polyamine). Other errors were caused by the lack of edges between candidates, which happened in some documents. In these cases, the PPR algorithm cannot be applied, and the candidate with the highest number of descendants is chosen, which is not always the correct choice and does not take into account the global coherence. Another common error is with chemical compounds that have different charges, for example, biliverdin and biliverdin(2-). These two concepts are linked by *is conjugate acid of* and *is conjugate base of* relations. However, they have a different set of *is-a* ancestors, having only organic molecular entity and its respective ancestors in common. More contextual information from the text could help understand the specific molecule that is being mentioned. Many entities of the gold standard were not annotated with ChEBI identifiers (Table 1). These missing identifiers could improve the results of our method since the graph of each document would be more exhaustive, and the global coherence score would take into account the complete set of entities. As both compounds appear in the same candidate list, the fact that the latter has a relation with the CHEBI:22563 (“anion”), a concept with many descendants, resulted in a higher score. In Fig. 1 we can see that “biliverdin(2-)” is one concept away from “organic anion”, which has 166 descendants.

On the HPO-GSC corpus, some errors were due to concepts with similar meanings. For example, “microretrognathia” and “micrognathia” are both facial deformations related to the development of the fetal mandible, and their respective HPO concepts have the same edges. Another common error was when dealing with child and parent concepts. For example, HP:0009588 refers to Vestibular Schwannoma and HP:0009589 to Bilateral vestibular Schwannoma and both appear in the candidate list for Bilateral vestibular Schwannoma. The parent concept, Vestibular Schwannoma, obtained a higher score, resulting in an error. Parent concepts are closer to the top concepts, and therefore it will have paths to more concepts. As it can be seen in Fig. 1a, HPO has several instances where related concepts have similar labels, with a difference of just one word. Even though we try to account for this issue by giving more weight to concepts with higher information content, sometimes this weight is not enough and concepts that have more links are ranked higher than the correct candidate. As the nomenclature of phenotypes is not as systematic as the nomenclature of chemical compounds, it is harder to perform entity linking on this domain, resulting in lower accuracy scores.

On both CRAFT-BP and CRAFT-CC corpora, some common errors occurred when dealing with child and parent concepts, just like it had been previously described on the HPO-GSC corpus. However, normally the candidate with higher IC was picked, as this is a factor in the calculation of the coherence score (Eq. 12). For example, RNA metabolic process (GO:0016070) and metabolic process (GO:0008152) were on the candidate list for metabolic (GO:0008152) and the chosen candidate was the more informative concept RNA metabolic process, which is not the correct candidate. Neither of the concepts had links with other candidates, so it was chosen the concept that had higher IC, RNA metabolic process.

Another aspect to highlight on the CRAFT-BP and CRAFT-CC corpora, although not the focus of the present study, is the high recall achieved while performing disambiguation: 0.77295 and 0.84315, respectively. Comparing with the values referred in [37], where the recall was less than 0.20 for CRAFT-BP and less than 0.70 for CRAFT-CC, the results obtained are a noticeable improvement, even more if we consider that the precision (or accuracy) has not been negatively affected.

## Conclusion

Entity linking is an essential task in text mining systems so that the information extracted can be linked to existing resources. However few approaches take advantage of the extensive knowledge encoded in domain-specific ontologies. We proposed a method, PPR-SSM, that combined existing entity linking graph-based approaches with semantic similarity to calculate a global coherence score. Using this score, we select the best candidate matches to a named entity. Our method outperformed string matching and PPR-based methods in four case-studies, obtaining an accuracy of 0.8039 on the ChEBI-patents gold standard, 0.6825 on HPO-GSC, 0.8244 on CRAFT-BP and 0.7258 on CRAFT-CC. These results show the potential of the proposed method to be adapted to other domains where ontologies are available. The code used to implement the method is publicly available^2^.

As future work, we could take advantage of the similarities between ontologies to develop a joint approach, as suggested by [38]. The authors of this paper have shown that considering multiple ontologies together is beneficial to biomedical EL. Other improvements would be to improve the candidate generation process, so that more entities have the correct candidate in their candidate list. One possible approach would be to use word embeddings to find the most similar concepts, instead of matching the characters of the string. Furthermore, the document graph could be improved using relation extracted from the document between entities. This way, if a relation between two entities is stated in the document, but not in the ontology, we could still use that information.

## Methods

### Problem definition

We now define the concepts necessary to understand the entity linking problem and our proposed solution. We consider the problem setting where a corpus of documents is annotated with entity mentions, and each entity mention has a set of KB candidate matches. The objective of entity linking is to link each entity mention to an entry of a KB. We can define a KB as a tuple <*C*,*R*>, where C is the set of concepts about a particular subject, and R the set of relations between the concepts, where each relation is a pair of concepts (*c*_1_,*c*_2_) with *c*_1_,*c*_2_∈*C*^3^. We consider a candidate list $CL(e) = \{ c_{e}^{1},..., c_{e}^{i} \} $ for each entity *e*∈*E*, where *E* is the set of named entities mentioned in a document. We want to find the *c*_*e*_∈*CL*(*e*) that best represents each *e*.

For each document, we can construct a graph *G* consisting of the edges defined by:

$$G = \{ (e, c_{e}) | e \in E, c_{e} \in CL(e) \} $$

where *e* corresponds to each named entity of a document and *c*_*e*_ to each candidate match of that entity. Hence, each node of *G* represents a candidate to a given entity of the document. The entities themselves are not represented in the graph, only the candidate concepts associated with them. However, different entities may have common candidate concepts, which would be represented as different nodes in the graph.

Our objective is to define a function *disambiguate* such that

$$disambiguate(e) = \operatorname*{arg\,max}_{c_{e}}\{ score(e, c_{e})\} $$

where *score* is a scoring function that evaluates how likely the candidate is to be the correct choice for entity *e*.

### Ontology-based personalized pageRank

We assume that a measure of global coherence among the candidate concepts could be used as a scoring function. The coherence of a candidate concept quantifies how well it fits among a set of concepts, while the global coherence estimates how well a set of candidate concepts fit with each other. This idea has been explored by other authors, who suggest random walks methods such as PPR to rank the importance of each node in a graph. Nodes with greater weight would be more relevant to the results. The weights are determined by simulating random walks on the graph, with a certain probability of jumping to a random node. The PPR algorithm is a variant of PageRank where the jump is always made to the same node. Using the graph previously described, we apply the PPR algorithm to calculate the weights of each node in relation to each other, which we use as a coherence score. While Pershina et al. [25] use a general purpose KB to run the PPR algorithm, we use domain-specific ontologies, which usually contain more detailed nodes and edges.

Note that in our graph model, each node represents a candidate concept associated with a named entity. Therefore, we consider only edges between nodes associated with different entities, since only one element of each candidate list can be correct. Our approach to entity linking explores the structure of the ontology to generate the graph. If a node is within distance *d* of another node, we consider that they are linked. To calculate this distance, we do not take into consideration the directionality of the relations of the ontology. Therefore, any two nodes of the same document can form an edge as long as there is a path with length equal to or shorter than *d* between them, and they are associated with different entities.

**Fig. 5:**
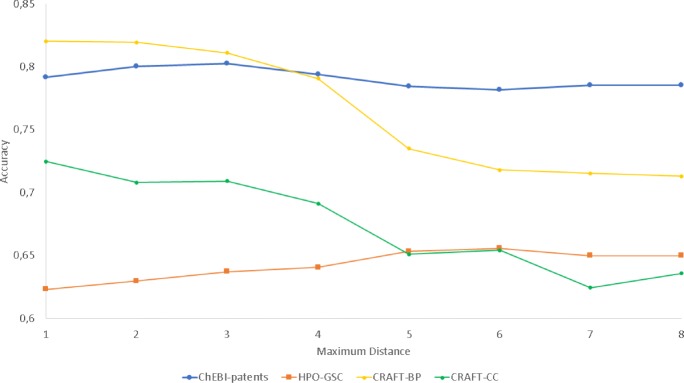
Example of the graph generated from abstract PMID2888021 using HPO. We show two candidate matches for each entity mention, and the edges obtained from the HPO ontology between each matches. Each candidate match is represented by its ontology ID and its preferred label

Figure 5 shows an example of the graph generated by a set of named entities from an abstract annotated with HPO concepts. To simplify the figure, we show only three entities and the two highest scoring candidates of each entity. We considered *d*=6 for this example. Due to its spelling similarity, tremor is a candidate match to the entity “tumour”, when in fact the correct match should be neoplasm. Note that neoplasm is a candidate for that entity because HPO has tumour as a synonym of neoplasm. The candidate tremor is linked only to one other candidate, while tumour is linked to candidates from both entities. Hence, neoplasm is more likely to maximize the global coherence. Likewise, Abnormality of the nervous system is linked only to one candidate, so it will have a negative contribution to the global coherence. Both candidates of the entity “neurofibromatosis” are linked to the same concepts. In these cases, we adopt a conservative approach and pick the candidate with more descendants in the ontology, since it represents a more generic concept. Therefore, neurofibromas would be the chosen candidate for that entity.

The PPR algorithm is used to calculate the coherence of each node in relation to another node, which can also be interpreted as the PageRank score. To accomplish this, we personalized the graph to each node, referred to as the source node. We estimate the coherence of node *n* to source node *s*, given by *PPR*(*s*→*n*), corresponding to the weight of *n* when personalizing to *s*. We multiply the PPR score by the normalized IC value of the concept associated with node *n*, in order to account for the different degrees of specificity of the concepts of an ontology. Therefore we calculate the coherence of node *n* relative to node *s* as

1 $$  coherence_{s}(n) = PPR(s \rightarrow n) \cdot IC(n)  $$

We estimate IC of a node *n* as:

2 $$  IC(n) = -\log(p(n))  $$

where *p*(*n*) is the probability of that node appearing on a corpus [39].

Finally, we sum the coherence score of node *n* to each source node *s* to estimate its global coherence:

3 $$  coherence(n) = \sum_{s \in G} coherence_{s}(n)  $$

### Semantic similarity

Semantic similarity measures (SSM) estimate the similarity between concepts using the relations defined by an ontology [40]. We explore how taking into account the semantic similarity between concepts can improve the graph model previously described, by adjusting the contribution of each node to another node. If two nodes share more semantics, they should have a greater contribution to each other’s global coherence score.

SSMs are normally restricted to subsumption relations (*is-a* or *subClassOf*), which are transitive, meaning that if *R* is the set of relations between concepts, (*c*_1_,*c*_2_)∈*R*, and (*c*_2_,*c*_3_)∈*R*, then (*c*_1_,*c*_3_)∈*R*. Therefore, the ancestors of *c* are given by

4 $$\begin{array}{*{20}l} Anc(c) = \{a : (c,a) \in T\} \end{array} $$

where *T* is the smallest relation set on *C* that contains *R* and is transitive.

Many SSMs explore the ancestors exclusive to each concept, as well as their common ancestors. We can define the common ancestors *CA* between two concepts as

5 $$\begin{array}{*{20}l} CA(c_{1}, c_{2}) = Anc(c_{1}) \cap Anc(c_{2}) \end{array} $$

Some SSMs use only the Most Informative Common Ancestors (MICA), which can be considered the most relevant to compare entities:

6 $$  MICA(c_{1}, c_{2}) = \{ a : a \in CA(c_{1}, c_{2}) \wedge IC(a) = \\ \max\{IC(a) : a \in CA(c_{1}, c_{2})\}\}  $$

Alternatively, SSMs can consider multiple inheritance relations, which we refer to Disjunctive Common Ancestors (DCA):

7 $$  DCA(c_{1}, c_{2}) = \{a : a \in CA(c_{1}, c_{2}) \\ \wedge \forall_{a_{x} \in CA(c_{1}, c_{2})}PD(c_{1},c_{2},a) = \\ PD(c_{1}, c_{2}, a_{x}) \Rightarrow IC(a) > IC(a_{x}) \}  $$

where *PD* is a function that calculates the difference between the number of paths of *c*_1_ and *c*_2_ to their common ancestors.

SSMs can use the IC of the concepts to estimate its similarity. Several measures have been proposed, one of the most commonly used being the measure proposed by Resnik [39]:

8 $$  SSM_{Resnik}(c_{1},c_{2}) = IC_{shared}(c_{1}, c_{2})  $$

where *IC*_*shared*_ is the average of the information content of the MICA or DCA.

Another SSM was proposed by Lin et al. [41], which balances the IC of the common ancestors with the IC of the concepts themselves:

9 $$  SSM_{Lin}(c_{1},c_{2}) = \frac{2 \times IC_{shared}(c_{1}, c_{2})}{IC(c_{1}) + IC(c_{2})}  $$

Finally, Jiang and Conrath [42] proposed a measure of distance between concepts of an ontology, given by

10 $$ dist_{jc}(c_{1}, c_{2}) = IC(c_{1}) + IC(c_{2}) - \\ 2 \times IC_{shared}(c_{1}, c_{2})  $$

As an SSM should be inversely proportional to the distance (i.e. less distance, more similar), we can use this distance to calculate a semantic similarity score:

11 $$  SSM_{jc}(c_{1},c_{2}) = \begin{cases} \frac{1}{dist(c1,c2)}, \text{if}\ dist > 0 \\ 1,\text{otherwise} \end{cases}  $$

Each of the presented measures uses the IC of the common ancestors between the two concepts. As such, they can use either MICA or DCA to calculate the *IC*_*shared*_ factor. We adapted the coherence score of node *e* according to source node *s* as:

12 $$  coherence_{e} = PPR(s \rightarrow e) \cdot SSM(s, e) \cdot IC(e)  $$

where SSM corresponds to one of the three SSM previously described.

### Models

We studied the effect of SSMs as a factor on the scoring function, and how it affects the accuracy of entity linking results. We first applied a baseline approach that consisted in selecting the ontology concept label most similar to the textual entity mention. This was implemented using the Levenshtein distance to obtain the label with the shortest distance to the text. This approach compares only the lexical form of the label, ignoring any context and semantics.

Then, we applied the PPR algorithm, using an approach similar to [25], but adapted to biomedical ontologies, which we refer to as the PPR model. As shown in (1), we can adjust the PPR score of each node with its IC. We refer to this model as PPR-IC. As previously explained, our adaptation of this approach has a distance parameter, corresponding to the maximum ontology distance between concepts. We studied the effect of this parameter on the PPR algorithm, to find the best value to use for further experiments.

We can then further adjust the contribution of each node to another node in the graph with the semantic similarity between them. As opposed to the model proposed by Pershina et al., we use all the candidates associated with the other entity mentions and not just the top scoring. This SSM factor will increase the weight of similar concepts, most likely to be coherent with the source node, and reduce the contribution of concepts less related to the source node. We refer to this model as PPR-SSM and study the effect of three SSMs on the accuracy of entity linking. Furthermore, we compare two versions of each SSM: one using the IC of the MICA (6) and another using the DCA (7).

## Data Availability

The data and code used for this study are available at https://github.com/lasigeBioTM/PPRSSM.

## Abbreviations

BP: Biological process
CC: Cellular component
ChEBI: Chemical entities of biological interest
DCA: Disjunctive common ancestors
GO: Gene ontology
HPO: Human phenotype ontology
IC: Information content
JC: Jiang and conrath
KB: Knowledge base
MF: Molecular function
MICA: Most informative common ancestor
NER: Named entity recognition
PPR: Personalized pageRank
SSM: Semantic similarity measures

## Footnotes

1 https://github.com/lasigeBioTM/PPRSSM

2 https://github.com/lasigeBioTM/PPRSSM

3 We use typewriter to indicate a concept of a KB, italics to indicate relations between concepts, and quotes to indicate entity mentions.
